# Efficacy of a Fungal Formulation with the Nematophagous Fungus *Pochonia chlamydosporia* in the Biological Control of Bovine Nematodiosis

**DOI:** 10.3390/pathogens11060695

**Published:** 2022-06-16

**Authors:** Júlia dos Santos Fonseca, Vinícius Monteiro Ferreira, Samuel Galvão de Freitas, Ítalo Stoupa Vieira, Jackson Victor de Araújo

**Affiliations:** 1 Department of Veterinary Medicine, Federal University of Viçosa (UFV), Viçosa 36570-900, MG, Brazil; samuel.freitas@ufv.br (S.G.d.F.); jvictor@ufv.br (J.V.d.A.); 2 Department of Animal Parasitology, Federal Rural University of Rio de Janeiro (UFRRJ), Seropédica 23897-000, RJ, Brazil; viniciusmferreira@ufrrj.br; 3 Department of Veterinary Medicine, Univértix, Matipó 35367-000, MG, Brazil; italostoupavieira@gmail.com

**Keywords:** helminthosis, helminths, parasitic nematodes

## Abstract

In the control of bovine worms, biological control by nematophagous fungi stands out, especially *Pochoniachlamydosporia* which causes the destruction of helminth eggs. This study aims to test the effectiveness of a formulation containing the nematophagous fungus *Pochonia chlamydosporia* isolated for the biological control of bovine nematodiosis. Twelve cattle were divided into two groups: control group (GC) and the group that received the formulation (GT). Feces and pasture samples were collected for the research of gastrointestinal nematodes. Lung worms and trematodes were investigated. The animals were weighed monthly. The averages of temperature and rainfall were recorded. The supply of the fungus *Pochonia chlamydosporia* was not effective in reducing the eggs per gram of feces of gastrointestinal nematodes (EPG) of the animals, not differing statistically (*p* > 0.05) between the groups. The mean values of larvae recovered in the pasture did not differ significantly (*p* > 0.05). The genus *Haemonchus* sp. was the most prevalent. There was no correlation between the number of larvae with temperature and rainfall (*p* > 0.05). There was a statistically significant difference (*p* < 0.05) in the penultimate weighing of the experiment. The formulation containing *Pochonia chlamydosporia* was not efficient in the biological control of bovine gastrointestinal nematodes.

## 1. Introduction

Bovine verminosis is one of the main causes of losses in livestock. Cattle farming is harmed by impacts such as production losses, expenses with prophylactic and curative treatments, and mortality in the herd in extreme cases. In this scenario, some genera of greater importance and prevalence of gastrointestinal parasitic nematodes in cattle raised in tropical countries stand out, such as *Cooperia*, *Haemonchus*, and *Oesophagostomum*. The species *Dictyocaulus viviparus* is important in the parasitism of the lungs of these animals [1] along with the trematodes, whose species of importance in cattle are: *Fasciola hepatica* and *Eurytrema coelomaticum*, which are liver and pancreatic duct parasites of cattle, respectively [2,3].

For an efficient parasite control, it is necessary to know the epidemiology of the parasites. Using this knowledge, prophylactic measures can be used for effective control, reducing the use of chemicals, cases of anthelmintic resistance, and dependence on the use of anthelmintics, when associated with other control methods. One of these methods is biological control by nematophagous fungi [4].

Nematophagous fungi are present in the environment and their action takes place in the animal’s feces in the soil. Among the mechanisms of action, the destruction of free-living helminth eggs or larvae stands out. Among the species of fungi used for biological control, there is the ovicidal species *Pochonia chlamydosporia* that acts by causing the destruction of eggs and *Duddingtonia flagrans*, a larvicidal species that causes the destruction of helminth larvae [5]. In cattle farming, one method for the distribution of these fungi in the environment is the administration of fungal structures in the cattle diet [6].

*Pochonia chlamydosporia* is widely used as a biocontrol agent [7]. Its ovicidal action consists of the production of proteases (extracellular enzymes) that have ovicidal activity against helminth eggs in the feces environment. In addition, this fungal species can produce enzyme extracts against solid substrates such as proteins, lipids, and polysaccharides [8]. Morphological changes are caused in the shell and internal content of nematode eggs [7]. In addition, this species also produces secondary metabolites [9]. This species of fungus acts on parasite eggs of several species of domestic animals such as cattle, swine, horses, dogs, cats, and humans [5,10]. Many in vitro studies have shown the action of this fungus on eggs of *Eurytrema coelomaticum*, *Fasciola hepatica*, *Oxyuris equi*, *Ascaris suum*, *Ancylostoma* sp., among other diverse species [10], and also in vivo [5]. It is important to emphasize that there is no evidence in the literature of larvicidal action of this species. Although the species of fungus studied is ovicidal and the eggs of gastrointestinal nematodes are released in the animal’s feces, there is a very fast passage to hatching and entering the larval stage [10]. It is not yet known whether the fungus in question could influence the development of the larval stage.

The objective of this study was to test the effectiveness of a fungal formulation containing the nematophagous fungus *Pochonia chlamydosporia* isolated for the biological control of bovine nematodiosis in southeastern Brazil.

## 2. Results and Discussion

The supply of the fungus *Pochonia chlamydosporia* was not effective in reducing the eggs per gram of feces (EPG) of gastrointestinal nematodes of the animals. The mean values of EPG did not differ statistically (*p* > 0.05) between the two groups (Table 1). 

Due to the ovicidal action of the fungal species used in this experiment, these results may have occurred because of the rapid passage of eggs to the larval stage of gastrointestinal nematodes. The passage of gastrointestinal nematodes in the environment from the egg stage to hatching is rapid, and there may not have been enough time for the fungus to perform its ovicidal action.

Despite the results found here, the enzymatic mechanisms of these fungi in egg adhesion and consequent destruction are suggested by several studies [11]. Additionally, in vitro and in vivo studies have been developed to decrease the number of recurrent gastrointestinal nematode infections in domestic animals using ovicidal fungi [7]. However, the factors that often impair the effectiveness of these fungi are strategies developed by the parasites themselves, such as the rapid passage from the egg to larval stage mentioned above, which makes interaction with the egg difficult [10].

During the entire experimental period, no eggs of the Trematoda class and no larvae of the species *Dictyocaulus viviparus* were found. The experiment was conducted in the dry season which could have influenced the non-appearance of these organisms.

The mean values of infective larvae (L3) recovered from the pasture did not differ significantly (*p* > 0.05) between the treated and control groups (Table 2). These results could be explained by low precipitation rates, since, according to Silva [12], rain is necessary for the larvae to leave the fecal cake and migrate to the grass. In addition, in the dry period, the highest parasite load is concentrated in the animal and not in the pasture, due to the unfavorable environmental conditions of this period. Another factor that may explain the results found here is the penetration of L3 into the soil to avoid desiccation. All these factors may have interfered in the recovery of L3 by the technique used in this study and consequently in the results presented. 

The prevalence of helminths in coprocultures is shown in Table 3. *Haemonchus* sp. was the most prevalent, followed by *Cooperia* sp. and *Oesophagostomum* sp. In other studies, such as by Rodrigues et al. [13] and Vieira et al. [14] in southeastern Brazil, *Haemonchus* sp. was also the most prevalent.

**Figure 1:**
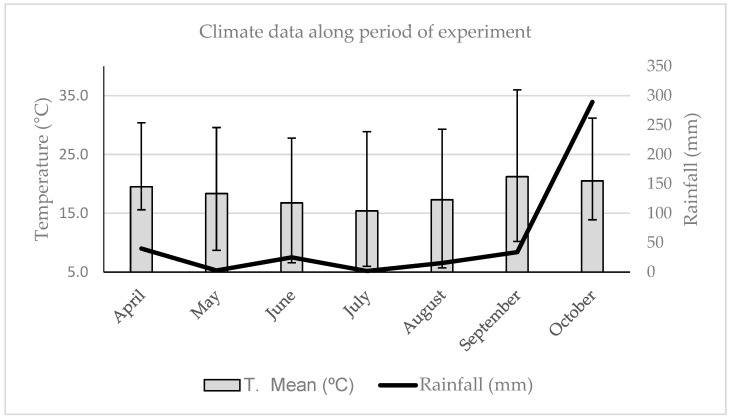
Minimum Temperature (T Min), Average (T Mean), Maximum (T Max), and Rainfall (mm/month) values from April to October 2021 in Viçosa, Minas Gerais, Brazil.

The climatic data recorded during the experimental period in the municipality of Viçosa (MG) are shown in Figure 1.

The highest averages of temperature recorded were in 30.4 °C (April), 36 °C (September), and 31.2 °C (October), at the beginning and end of the experimental period, respectively. The average temperatures recorded were 19 °C, 21 °C, and 20 °C in April, September, and October, respectively. In these months, the highest levels of rainfall were also recorded, with 39.8 mm in April, 33.8 mm in September, and 289.4 mm in October. It was observed that the months with higher temperatures also had the highest average rainfall. During the months of May to August, the lowest average indices of temperature and rainfall were recorded, characterizing the dry period. The minimum temperatures during these months were 8.7 °C in May, 6.6 °C in June, 6 °C in July, and 5.7 °C in August. The average temperatures were 18 °C in May, 17 °C in June, 15 °C in July, and 17 °C in August. The rainfall during this period was 2.6 mm in May, 25.00 mm in June, 1.8 mm in July, and 15.4 mm in August.

During the experimental period, there was no correlation between the number of larvae recovered with temperature and rainfall (*p* > 0.05). Thus, the meteorological data did not contribute to differences between the two experimental groups in this study.

This study was developed predominantly during the dry season of the year, with the lowest temperatures for this region and the lowest levels of rainfall, which may have contributed to the results found, since the evolutionary forms of helminths present in the environment are also disadvantaged by low temperatures and lack of rainfall in the dry period. The development and migration of infective forms are impaired [15]. Mkandawire et al. [16] reported that environmental factors directly influence the hatching of eggs. In addition, environmental factors, such as temperature, can interfere with the dynamics of nematode predation by fungi since each species has a different ability to adapt to climatic conditions [17]. Additionally, such factors impact the action of these fungi also because each fungal species has a different adaptive capacity [7].

The data of average daily weight gain in kilograms (kg/day) are presented in Table 4. There was no statistically significant difference (*p* > 0.05) from April to July and also at the last weighing of the experimental period, in the month of September. However, there was a statistically significant difference (*p* < 0.05) in the penultimate passage of the experiment in August, in the average of the entire experimental period, in which the weight of the animals in the group treated with the fungal formulation containing *Pochonia chlamydosporia* was higher than in the control group.

The statistically significant difference in the penultimate weighing of this study, in which greater weight gain was observed in the treated group compared to the control group, may indicate some action of the fungus. Oliveira et al. [18] also recorded an increase in weight gain in animals that received the fungus for six months compared to the control group, however, in the case of a larvicidal and non-ovicidal fungus.

For future studies, the associated use of the species *Pochonia chlamydosporia* with other important biocontrol fungi is important, with emphasis on the association between them and *Duddingtonia flagrans*. Some authors have shown that this association significantly increased predation when compared to the separate use of fungi, showing a synergistic effect between the fungi [19,20,21,22]. With these literature studies and the results of this work, the use of the fungus *Pochonia chlamydosporia* associated with a larvicidal fungus, such as *Duddingtonia flagrans*, may be a promising alternative for the future in the control of nematodiosis in cattle. The present results indicated that the formulation containing the *Pochonia chlamydosporia* isolate was not efficient in the biological control of bovine gastrointestinal nematodes.

## 3. Materials and Methods

The experiment was lead on a farm, situated in the city of Viçosa, MG, Brazil at a tropical climate in the dry season for 6 months, from April to October 2021. Twelve crossbred cattle, 18 months old, with an average weight of 300 kg were used for the experiment. Previously, the anthelmintic doramectina 1% (Dectomax^®^, Zoetis, São Paulo, Brazil) was administered at a dose of 1 mL/50 kg animal body weight in these animals. Fifteen days after treatment with the anthelmintic and when the absence of nematode eggs in the feces was confirmed, the animals were randomly divided into two groups with six animals each: control group (GC) and six animals that received the product formulation containing the fungus (GT). These were distributed in two paddocks with two hectares each of *Brachiaria decumbens*, naturally infested with helminth larvae, due to the previous grazing history of young animals.

The fungus tested was *Pochonia chlamydosporia* in a formulation conveyed in rice bran produced by Ghenvet Animal Health (Paulínia, SP, Brazil), containing 10^5^ chlamydospores per gram. In the GT, each animal was treated with 1 g (10^5^ chlamydospores) of *Pochonia chlamydosporia* for every 10 kg of animal live weight, administered daily together with commercial feed. In the GC, each animal received the same dosage, however, of the placebo (rice bran), that is without the fungus added to the ration.

During the six months of the experiment, every 15 days, after the animals were introduced to the pastures, stool samples were collected directly from the rectal ampulla of all animals in each group. In these samples, the counts of eggs per gram of feces (EPG) were determined by the method of Gordon and Whithlock [23] modified by Lima [24]. In addition to EPG, coprocultures were produced with 20 g of feces mixed with vermiculite and taken to an incubator at 26 °C for 15 days to obtain infective larvae (L3), which were later identified according to Keith [25]. For the research of lung worms, the method of Baermann [26] was used. For the search for trematodes, the method of Dennis et al. [27] was used.

Additionally, every 15 days, two samples of pasture (0–20 and 20–40 cm away from the fecal cake) were collected from the GT and GC paddocks in (W) from six alternate points, according to Raynaud and Gruner [28]. Samples of 500 g pasture were used to recover the L3 following the methodology described by Lima [24]. Subsequently, the sediment was examined under an optical microscope (Olympus Corporation, Tokyo, Japan) to identify and count the larvae according to the criteria established by Keith [25]. These samples were placed in an oven for three days at 70 °C to obtain dry matter (DM). The data obtained were transformed into the number of larvae per kilogram of DM.

The weight of the animals was taken monthly. Weight gain was calculated by subtracting current weight from previous weight and dividing by days between the two weighings. The result was given in kg/day. The averages of minimum, average, and maximum temperature and rainfall during the experimental period were recorded.

The results obtained were submitted to Student’s t-test at a significance level of 5%.

This experimental trial strictly followed all the recommended procedures by the rules of use in animals and certified by the Comitê de Ética de Uso Animal (CEUA/UFV). This project was approved by CEUA/UFV, file no. 42/2021.

## 4. Conclusions

The rapid transition from egg to larvae stage was not affected by the fungus *Pochonia chlamydosporia*, therefore the fungal formulation containing *Pochonia chlamydosporia* was not efficient in the biological control of bovine gastrointestinal nematodes.

## Figures and Tables

**Table 1:** Average values ± standard deviation (SD) of the eggs per gram of feces (EPG) values of gastrointestinal nematodes from animals in the group treated with the fungal *Pochonia chlamydosporia* and in the control group during the period from April to October 2021, in Viçosa, Minas Gerais, Brazil.

Month	EPG	Control	Treated
April	1°	50.0 (83.3) ^a^	83.3 (98.3) ^a^
	2°	133.3 (20.0) ^a^	20.0 (44.7) ^a^
May	3°	133.3 (233.8) ^a^	33.3 (81.6) ^a^
	4°	133.3 (175.1) ^a^	116.6 (132.9) ^a^
June	5°	66.7(121.1) ^a^	133.3 (186.2) ^a^
	6°	100.0 (167.3) ^a^	66.7 (121.1) ^a^
July	7°	50.0 (122.4) ^a^	50.0 (54.7) ^a^
	8°	100.0(167.3) ^a^	133.3 (196.6) ^a^
August	9°	16.7 (40.8) ^a^	50.0 (83.6) ^a^
	10°	33.3 (51.6) ^a^	16.7 (40.8) ^a^
September	11°	83.3 (204.1) ^a^	216.7 (278.6) ^a^
	12°	100.0 (200.0) ^a^	183.3 (183.4) ^a^
October	13°	83.3 (160.2) ^a^	233.3 (301.1) ^a^
	14°	150.0 (320.9) ^a^	16.7 (40.8) ^a^

Arithmetic means with the same letters do not differ significantly by the Student’s *t*-test (*p* > 0.05).

**Table 2:** Mean values of infective larvae (L3) of nematodes recovered per kilogram of dry matter ± standard deviation (SD) in the group treated with the fungus *Pochonia chlamydosporia* and in the control group during the period from April to October 2021, in Viçosa, Minas Gerais, Brazil.

T20	C20	T40	C40
24.9 (67.6) ^a^	21.5 (31.7) ^a^	13.3 (37.7) ^a^	9.44 (15.7) ^a^

Legend: 20 and 40 represent, respectively, the distance of 0–20 and 20–40 cm from the fecal cake at which the grass was collected from the paddocks of the treated and control groups. Arithmetic means with the same letters do not differ significantly by the Student’s *t*-test (*p* > 0.05).

**Table 3:** Mean values of the percentages of *Haemonchus* sp. (Haem), *Cooperia* sp. (Coop), and *Oesophagostomum* sp. (Oeso) of L3 of nematodes recovered from coprocultures of groups of animals treated with the formulation containing *Pochonia chlamydosporia* and the control group during the period from April to October 2021 in Viçosa, Minas Gerais, Brazil.

	Treated			Control	
Haem	Coop	Oeso	Haem	Coop	Oeso
54.4%	22.8%	22.8%	61.4%	23.9%	14.3%

**Table 4:** Average daily weight gain in kilograms (kg/day) ± standard deviation (SD) of young crossbred cattle from the control group and the group treated with a formulation containing the isolated fungus *Pochonia chlamydosporia*, from April to October 2021 in Viçosa, Minas Gerais, Brazil.

ADG	1°–2°	2°–3°	3°–4°	4°–5°	5°–6°	6°–7°
Treated	−0.50 (0.9) ^a^	1.08 (0.7) ^a^	0.24 (0.2) ^a^	−0.38 (0.8) ^a^	1.27 (1.0) ^a^	−0.03 (1.0) ^a^
Control	0.43 (1.0) ^a^	0.53 (1.2) ^a^	0.00 (0.7) ^a^	0.20 (0.3) ^a^	0.30 (0.6) ^b^	0.54 (0.3) ^a^

Arithmetic means with different letters differ significantly by Student’s *t*-test (*p* < 0.05).

## Data Availability

The data presented in this study are available upon request from the corresponding author.
